# ATP-Dependent C–F Bond Cleavage Allows the Complete Degradation of 4-Fluoroaromatics without Oxygen

**DOI:** 10.1128/mBio.00990-16

**Published:** 2016-08-09

**Authors:** Oliver Tiedt, Mario Mergelsberg, Kerstin Boll, Michael Müller, Lorenz Adrian, Nico Jehmlich, Martin von Bergen, Matthias Boll

**Affiliations:** a Faculty of Biology, Microbiology, Albert-Ludwigs-Universität Freiburg, Freiburg, Germany; b Institute of Pharmaceutical Sciences, Albert-Ludwigs-Universität Freiburg, Freiburg, Germany; c Department of Isotope Biogeochemistry Proteomics, Helmholtz Centre for Environmental Research, UFZ, Leipzig, Germany; d Department of Molecular Systems Biology, Helmholtz Centre for Environmental Research, UFZ, Leipzig, Germany; e Faculty of Biosciences, Pharmacy and Psychology, University of Leipzig, Leipzig, Germany; f Department of Chemistry and Bioscience, University of Aalborg, Aalborg, Denmark

## Abstract

Complete biodegradation of the abundant and persistent fluoroaromatics requires enzymatic cleavage of an arylic C–F bond, probably the most stable single bond of a biodegradable organic molecule. While in aerobic microorganisms defluorination of fluoroaromatics is initiated by oxygenases, arylic C–F bond cleavage has never been observed in the absence of oxygen. Here, an oxygen-independent enzymatic aryl fluoride bond cleavage is described during the complete degradation of 4-fluorobenzoate or 4-fluorotoluene to CO_2_ and HF in the denitrifying *Thauera aromatica*: the ATP-dependent defluorination of 4-fluorobenzoyl-coenzyme A (4-F-BzCoA) to benzoyl-coenzyme A (BzCoA) and HF, catalyzed by class I BzCoA reductase (BCR). Adaptation to growth with the fluoroaromatics was accomplished by the downregulation of a promiscuous benzoate-CoA ligase and the concomitant upregulation of 4-F-BzCoA-defluorinating/dearomatizing BCR on the transcriptional level. We propose an unprecedented mechanism for reductive arylic C–F bond cleavage via a Birch reduction-like mechanism resulting in a formal nucleophilic aromatic substitution. In the proposed anionic 4-fluorodienoyl-CoA transition state, fluoride elimination to BzCoA is favored over protonation to a fluorinated cyclic dienoyl-CoA.

## IMPORTANCE

Organofluorides are produced as pesticides, pharmaceuticals, and other chemicals and comprise approximately one quarter of all organic compounds in the pharmaceutical and agricultural sectors; they are considered a growing class of environmentally relevant persistent pollutants. Especially in the case of fluoroaromatics, biodegradation is hampered by the extreme stability of the arylic C–F bond. In aerobic microorganisms, degradation proceeds via oxygenase-dependent C–F bond cleavage reactions, whereas the enzymes involved in the degradation of fluoroaromatics at anoxic sites are unknown. Here we report a strategy for the complete biodegradation of a fluoroaromatic to CO_2_ and HF in a denitrifying bacterium via activation to a CoA ester, followed by oxygen-independent arylic C–F bond cleavage catalyzed by an ATP-dependent enzyme. This reaction, in conjunction with a transcriptional adaptation to fluorinated growth substrates, is essential for the anoxic biodegradation of 4-fluorobenzoate/4-F-toluene and probably other fluoroaromatics.

## INTRODUCTION

Today more than a million different chemicals and 20 to 30% of all commercially available pharmaceuticals and chemicals applied in agriculture contain one or more fluorine substituents (1–5). Incorporation of fluorine into organic compounds can considerably influence their stability, lipophilicity, and biological activity. Due to the similar steric properties of hydrogen and fluorine, fluorinated compounds are often cometabolized to hazardous dead-end metabolites (4, 6). Among the organofluorides, the fluoroaromatics are particularly recalcitrant because the C_6_H_5_-F bond is probably the strongest single bond of an organic molecule (bond dissociation energy DH_298_ ≈ 530 kJ mol^−1^) (7).

The capacity for the complete degradation of fluoroaromatics has frequently been reported for aerobic microorganisms, and most results derive from studies with fluorobenzoates as model compounds (for a recent review, see reference 8). Briefly, promiscuous ring-hydroxylating dioxygenases form fluorinated cyclohexadienediol carboxylic acids that, depending on the position of the fluorine, undergo different further conversions.

Under anoxic conditions, the transformation of halogenated aromatic compounds is mainly associated with organohalide respiration, in which the reductive cleavage of C–halide bonds is catalyzed by membrane-bound, corrinoid-containing reductases and coupled to energy conservation (9–11). In this process, aromatic organohalides serve as terminal electron acceptors of respiratory chains, and the dehalogenated aromatic products are not further degraded. Organohalide respiration has been reported for reductive dehalogenation of numerous organochlorides and -bromides, but never for an organofluoride. This finding can be rationalized by the extraordinary strength of the C–F bond of fluoroaromatics and/or by the strong negative partial charge of fluorine substituents as revealed by electron density modeling (12).

The complete anaerobic degradation of fluoroaromatics has been reported only for 2- and 4-fluorobenzoate (2-/4-F-benzoate) in a sulfate-reducing bacterium (13) and a few denitrifying bacteria (14–19). While the two fluorinated benzoates can be activated to the corresponding coenzyme A (CoA) esters by promiscuous conventional benzoate-CoA ligases (BCL) (20–22), enzymatic defluorination of a potential fluorobenzoyl-CoA (F-BzCoA) intermediate has never been demonstrated. Class I benzoyl-CoA reductase (BCR) catalyzes the electron donor- and ATP-dependent reduction of 2-F-BzCoA possibly to a fluorinated dienoyl-CoA; however, the nature of the substrate has not been determined (23). In contrast, 4-F-BzCoA was reported to act as an inhibitor rather than a substrate of BCR in *Thauera aromatica* K172 (*T. aromatica*) (24). In summary, the enzymatic processes involved in defluorination during growth with 2- and 4-F-benzoate have remained unknown.

The only insight into the complete biological degradation of an aromatic organohalide in the absence of oxygen has been obtained in studies of 3-Cl-benzoate degradation in the phototrophic *Rhodopseudomonas palustris* (25) and the denitrifying *Thauera chlorobenzoica* (26). In both facultative anaerobes, a specific 3-Cl-BCL has been characterized. In *T. chlorobenzoica*, 3-Cl-BzCoA has been shown to be converted to BzCoA and HCl by a promiscuous, ATP-dependent class I BCR using Ti(III) citrate as an artificial electron donor (26). This finding is remarkable, as the known cellular function of BCR has been considered to catalyze the ATP-dependent dearomatization of BzCoA to cyclohexa-1,5-diene-1-carboxyl-CoA (dienoyl-CoA), a key reaction in the anaerobic degradation of aromatic compounds (23, 27, 28). A two-step process has been proposed in which 3-Cl-BzCoA is reduced to 3-Cl-dienoyl-CoA, driven by ATP hydrolysis, followed by enzymatic or, more likely, spontaneous E2 elimination of HCl, driven by rearomatization to BzCoA. The latter is then ATP-dependently dearomatized by the same reductase. Notably, 3-F-BzCoA was not dehalogenated by class I BCR but was rather converted to 3- and/or 5-F-dienoyl-CoA dead-end products.

Here, we investigated the processes involved in enzymatic defluorination in *T. aromatica* during anaerobic growth with 4-F-benzoate/4-F-toluene as fluoroaromatic model growth substrates. We identified a previously unknown mode of enzymatic dehalogenation, including the activation to 4-F-BzCoA and the ATP-dependent reductive cleavage of the arylic C–F bond by a formally nucleophilic aromatic substitution; adaptation to growth with 4-F-benzoate was accompanied by the concomitant down- and upregulation of 4-F-BCL and 4-F-BCR activities.

## RESULTS

### Growth of *T. aromatica* with 4-F-benzoate and 4-F-toluene.

**FIG 1:**
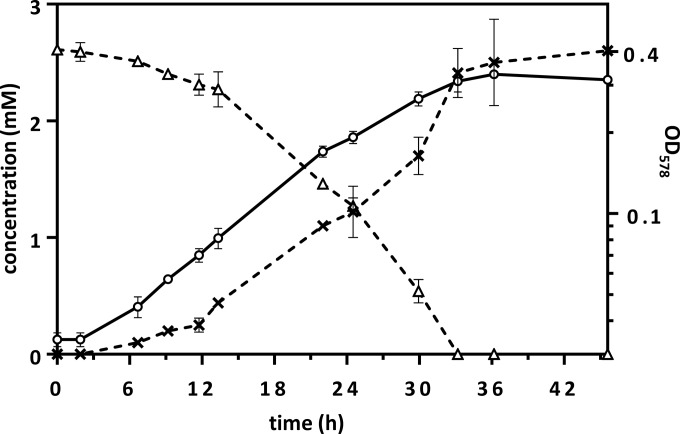
Growth of *T. aromatica* K172 with 4-F-benzoate. The OD_578_ (○) and concentrations of 4-F-benzoate (△) and fluoride (×) are shown. The means of two biological replicates are shown.

The degradation of 4-F-benzoate in *T. aromatica* was chosen as a model system to elucidate the unknown enzymatic processes involved in the complete anaerobic degradation of fluoroaromatics. In previous work, cell suspensions of *T. aromatica* K172 and other strains of the genera *Thauera* and *Azoarcus* have been reported to transform some halogenated benzoates, including 4-F-benzoate, but no correlation of growth and fluoride release has been demonstrated (16, 17). We investigated 4-F-benzoate degradation by cultivating *T. aromatica* in a mineral medium containing 2.7 mM 4-F-benzoate as the only carbon source and 12.7 mM nitrate as the electron acceptor. A time-dependent increase of cell density that was accompanied by a stoichiometric 4-F-benzoate consumption and fluoride formation was observed (Fig. 1); no growth was observed when 4-F-benzoate was omitted from the assay. With an optical density at 578 nm (OD_578_) of 1.0 corresponding to 0.22 g of cells (dry weight) liter^−1^ (our determination), the growth yield was ≈35 g of cells (dry weight) per mol 4-F-benzoate consumed; it remained constant during the exponential growth phase (see Table S1 in the supplemental material). The doubling time was ≥9 h, which was four times higher than that with benzoate.

4-F-toluene was tested as an additional growth substrate for *T. aromatica* that may be degraded via intermediates of the 4-F-benzoate degradation pathway. A substrate-dependent increase of cell density and fluoride release to the medium was observed; the doubling time was 16.2 h, which was 3.3-fold higher than with toluene (see Fig. S1 in the supplemental material). Growth with both 4-F-toluene and 4-F-benzoate suggests that the former may be converted by promiscuous enzymes of the benzylsuccinate degradation pathway to a common 4-F-BzCoA intermediate (29).

### Whole-cell suspension assays.

**FIG 2:**
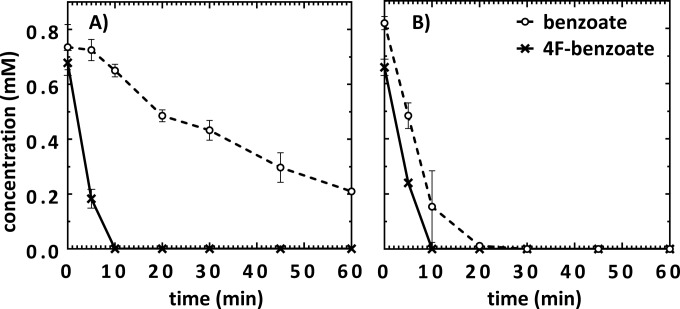
Consumption of benzoate and 4-flurobenzoate by cell suspensions of *T. aromatica*. Suspensions of cells grown with benzoate (A) or grown with 4-F-benzoate (B). The means of two biological replicates are shown.

To test whether growth with 4-F-benzoate requires the induction of new and/or additional enzymes, substrate consumption studies using a dense suspension (OD_578_ ≈ 9) generated from cells grown with 4-F-benzoate and benzoate as a control were performed. Benzoate-grown cells readily consumed 0.75 mM benzoate within 10 min, whereas the rate of 4-F-benzoate consumption was significantly decreased to approximately 5 to 10% compared to that of benzoate consumption (Fig. 2A). In comparison to benzoate-grown cells, consumption of 4-F-benzoate was clearly stimulated in 4-F-benzoate-grown cells (Fig. 2B). This finding strongly suggests that the synthesis of the enzymes involved in 4-F-benzoate catabolism is induced during growth with the fluorinated growth substrate.

### *In vitro* assays for initial enzymatic steps involved in 4-F-benzoate degradation. 

Isolated BCL (AMP forming) from *T. aromatica* has been reported to readily convert all three monofluorinated benzoate analogues to their corresponding CoA esters, with 4-F-benzoate and benzoate being activated at almost identical rates (20). We investigated 4-F-BCL/BCL activity in extracts of *T. aromatica* cells grown with 4-F-benzoate after ammonium sulfate fractionation; the latter step was necessary to remove background activities disturbing the coupled spectrophotometric assay (e.g., unspecific NADH oxidation, ATP or CoA ester hydrolysis). In agreement with previous studies, both 4-F-benzoate and benzoate were converted by cell extracts at nearly identical rates.

**FIG 3:**
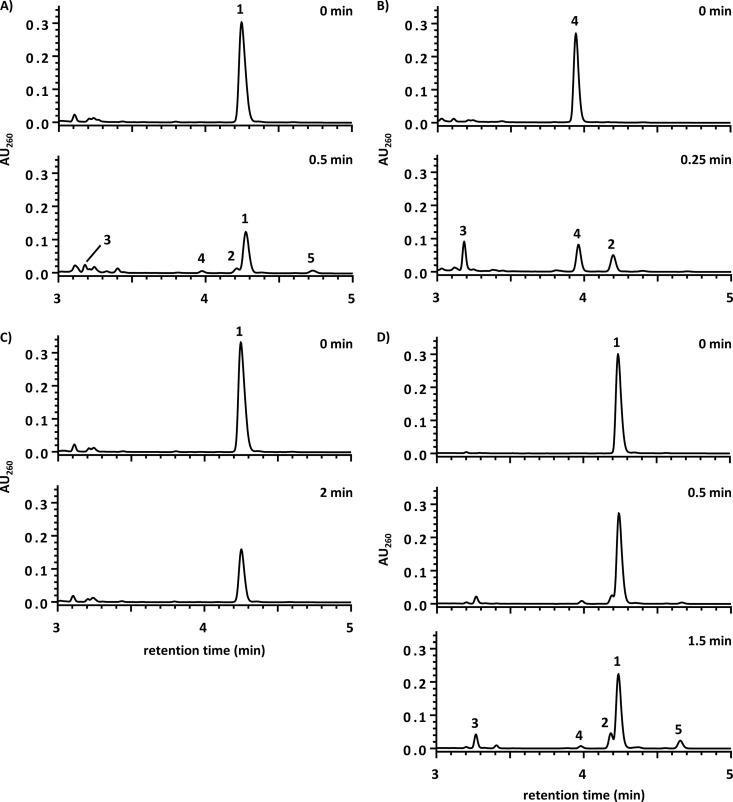
Selected HPLC diagrams demonstrating the conversion of aromatic CoA esters by cell extracts of *T. aromatica* grown with 4-F-benzoate (A to C) and by purified 4-F-BCR (D). All assays contained Ti(III) citrate (5 mM) and 0.2 mM of the individual CoA ester substrates. Conversion of CoA esters by cell extracts: 4-F-BzCoA in the presence of MgATP (A), BzCoA in the presence of MgATP (B), 4-F-BzCoA without MgATP (C), 4-F-BzCoA by purified 4-F-BCR (contaminated by cyclohexa-1,5-diene-1-carboxyl-CoA hydratase) (D). The loss of peak areas was due to thioesterase activity of the extracts that was subtracted from the activities determined. Compounds are indicated in the figure by numbers above the peaks as follows: 1, 4-F-BzCoA; 2, 1,5-dienoyl-CoA; 3, 6-OH-monoenoyl-CoA; 4, BzCoA; 5, 1-monoenoyl-CoA. An additional minor peak eluting at 3.4 min in panels A and D is assigned to 2-hydroxycyclohexanecarboxyl-CoA, which is formed from compound 5 by a side reaction of 1,5-dienoyl-CoA hydratase present in cell extracts and as a highly active minor contamination in purified BCR. AU_260_, relative absorption units at 260 nm.

Using anaerobically prepared extracts from *T. aromatica* grown with 4-F-benzoate, the time- and protein-dependent consumption of 4-F-BzCoA and formation of several products was observed (Fig. 3A). Conversion of 4-F-BzCoA was strictly dependent on both Ti(III) citrate (not shown) and MgATP (Fig. 3C). The products formed from 4-F-BzCoA were identified by coelution with standards and by electrospray ionization (ESI)/mass spectrometric analysis as follows (compound numbers refer to the numbers above peaks in Fig. 3): BzCoA (*m/z* = 872.6; compound 4), 6-hydroxycyclohex-1-ene-1-carboxyl-CoA (6-OH-monoenoyl-CoA; *m/z* = 892.6; compound 3), cyclohexa-1,5-diene-1-carboxyl-CoA (1,5-dienoyl-CoA; *m*/*z* = 874.5; compound 2), and cyclohex-1-ene-1-carboxyl-CoA (1-monoenoyl-CoA; *m/z* = 876.3, compound 5). The products compounds 2 to 4 are typical intermediates of the BzCoA degradation pathway as demonstrated in an assay with BzCoA instead of 4-F-BzCoA as the substrate (Fig. 3B). The rather artificial accumulation of the cyclic monoenoyl-CoA compound 5 is a result of dienoyl-CoA reduction and has frequently been observed during accumulation of the components compounds 2 and 3 in the reaction assay (30). Most importantly, no fluorinated CoA ester other than 4-F-BzCoA was identified, indicating a quantitative 4-F-BzCoA defluorination into BzCoA in an ATP-dependent manner. The specific 4-F-BzCoA consumption activity in extracts from cells grown with 4-F-benzoate varied between 3 and 5 nmol min^−1^ (mg protein)^−1^ but was constantly 6% ± 3% (mean value of triplicate determinations ± standard deviation) of the BzCoA conversion rate (50 to 80 nmol min^−1^ mg^−1^).

### Identification of BCR as 4-F-BzCoA-defluorinating enzyme. 

**FIG 4:**
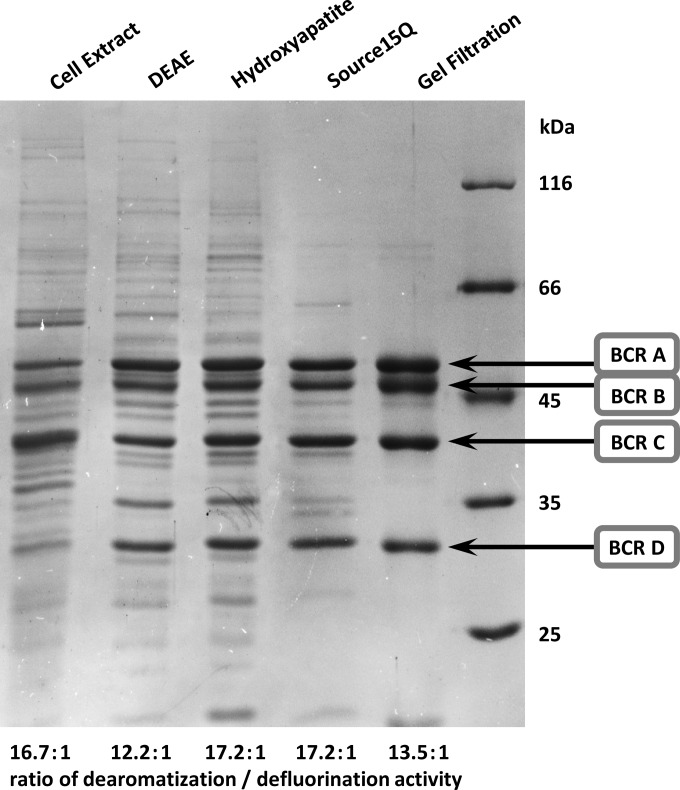
Purification of 4-F-BzCoA reducing activity and identification of 4-F-BCR as BCR. The highly enriched enzyme after gel filtration was composed of the four subunits of class I BCR (BcrABCD). The BzCoA-dearomatizing activity and 4-F-BzCoA-defluorinating activity coeluted after all purification steps with an almost constant ratio of dearomatizing/defluorinating activity as indicated by the values presented below the gel.

The 4-F-BzCoA-defluorinating enzyme was enriched from extracts of cells grown with 4-F-benzoate and nitrate. Based on the protocol established for purification of BCR (23), three chromatographic steps and one gel filtration step were applied. Fractions obtained after each enrichment step were screened for 4-F-BCR and BCR activity using a spectrophotometric assay in which reduced methyl viologen replaced Ti(III) citrate as an artificial electron donor. Four protein bands were highly enriched at almost equal intensities (Fig. 4) that were identified as the four subunits of BCR (BcrABCD, gi:3724168 to 3724171). Most importantly, BCR and 4-F-BCR activities coeluted in each fraction during the entire enrichment procedure with only minor fluctuation of the relative activities as indicated in Fig. 4. The *K_m_* values of the enriched enzyme were 97 µM (4-F-BzCoA) and 43 µM (BzCoA). The latter value is close to the recently reported *K_m_* value (37 µM) (24). The defluorinating activity was strongly oxygen sensitive: incubation in air for 10 min almost completely abolished the activity, which is in accordance with the reported oxygen sensitivity of BCR (23).

### Differential activity, gene expression, and protein abundance of BCL and BCR. 

**FIG 5:**
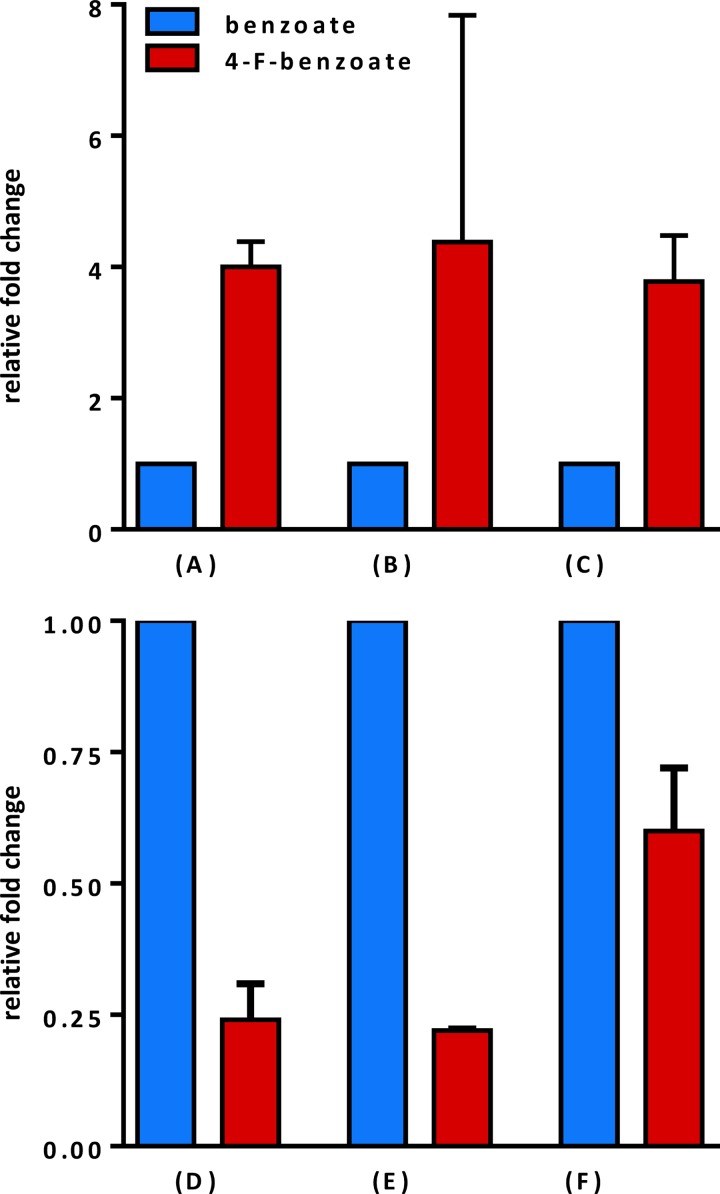
Differential regulation of BCL and BCR during growth with 4-F-benzoate versus benzoate. (A) Relative BCR activities, (B) relative abundances of Bcr(ABCD) (average abundance of all four subunits), (C) relative *bcr*(*ABCD*) transcript abundances (average abundance of all four genes), (D) relative BCL activities, (E) relative BCL subunit abundances, and (F) relative *bcl* transcript abundances. The error bars indicate standard deviations determined from three biological replicates.

The specific activities as well as the protein and gene transcript abundance of BCR and BCL were compared in extracts of cells grown with 4-F-benzoate and benzoate. Surprisingly, BCL activity was almost fourfold higher in extracts from cells grown with benzoate (150 nmol min^−1^ mg^−1^) than in cells grown with 4-F-benzoate (43 nmol min^−1^ mg^−1^; Fig. 5A), clearly suggesting a downregulation in the presence of the fluorinated aromatic carbon source versus nonfluorinated aromatic carbon source. The opposite effect was observed with BCR activity, which was almost fourfold higher in cells grown with 4-F-benzoate (50 to 80 nmol min^−1^ mg^−1^) versus benzoate (15 to 20 nmol min^−1^ mg^−1^; Fig. 5D).

The observed differential enzyme activities were further investigated with respect to the abundance of the individual proteins by comparative analysis of the proteomes of cells grown with benzoate or 4-F-benzoate. Peptide analyses were performed by a nanoscale liquid chromatography coupled to tandem mass spectrometry (nano-LC-MS/MS) (Orbitrap Fusion mass spectrometer) as described previously (31). In cells grown with 4-F-benzoate, BCL (gi:23450983) was found fourfold less abundant than in cells grown with benzoate (*P* < 5.3 × e^–11^; Fig. 5E). In contrast, the averaged abundance of the four subunits of BCR (BcrABCD) was around fourfold higher in 4-F-benzoate-grown cells (*P* < 3.5 × e^–5^; Fig. 5B). In summary, the data obtained from proteome analyses suggest that differential activities of BCL and BCR can be explained by their differential abundance in the cell (for a summary of protein identifications, see Table S2 in the supplemental material).

To test whether the observed differential abundance of BCL/BCR was due to a differential expression of the protein-encoding genes, quantitative PCR (qPCR) analyses of the individual protein-encoding genes was carried out. For this purpose, cells were harvested at different time points in the exponential growth phase, and the relative amounts of mRNA transcripts of the transcript-encoding *bcl* and *bcrABCD* genes were determined. The results obtained followed the tendency observed in *in vitro* assays and proteome analyses: the *bclA* gene transcript was less abundant in cells grown with 4-flurobenzoate versus benzoate, whereas the opposite was observed with the averaged abundance of the *bcrABCD* gene transcripts (Fig. 5C and F).

In summary, the results obtained provide evidence for a downregulation of BCL and upregulation of BCR on the transcriptional level as an adaptive response to growth with 4-F-benzoate versus benzoate.

## DISCUSSION

In this work, a previously unknown mode of enzymatic C–F bond cleavage was identified, catalyzed by ATP-dependent BCR from *T. aromatica*. It represents the so far only known enzymatic defluorination process allowing the anoxic utilization of fluoroaromatics as growth substrates. The ATP-dependent conversion of 4-F-BzCoA to BzCoA and HF is a newly identified activity of class I BCRs that expands the growth substrate range of aromatic-compound-degrading anaerobes to fluoroaromatics such as 4-F-benzoate and 4-F-toluene and probably other fluoroaromatics. During 4-F-toluene degradation, conversion to 4-F-BzCoA is expected to be catalyzed by promiscuous enzymes of the benzylsuccinate degradation pathway (29).

**FIG 6:**
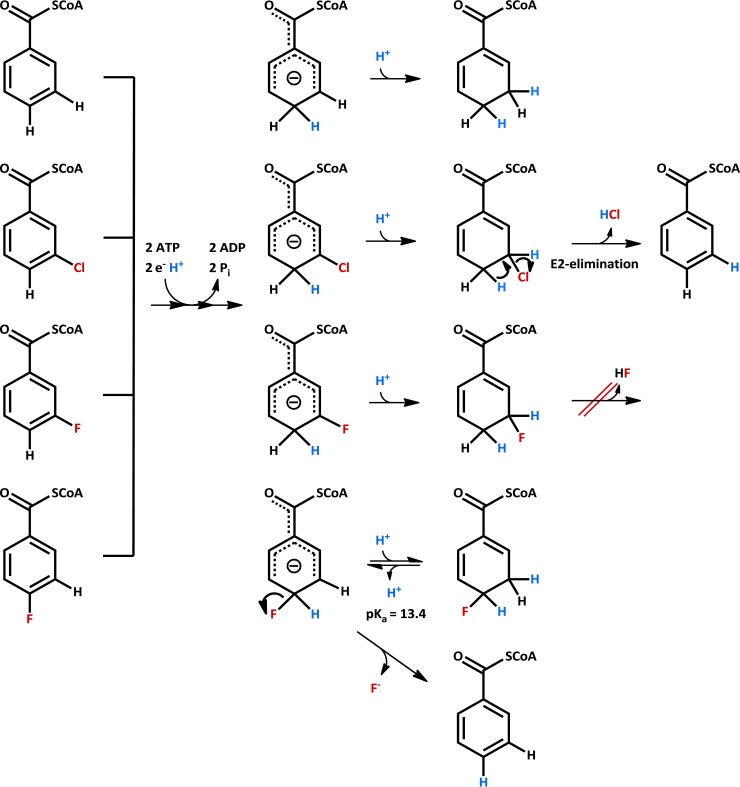
Possible mechanisms for the conversion of BzCoA and halogenated analogues by ATP-dependent class I BCR. A Birch-like mechanism involving two single ATP-dependent electron transfer steps and one protonation step yielding an anionic state is suggested for all shown BzCoA analogues. In the cases of BzCoA, 3-Cl-BzCoA, and 3-F-BzCoA, protonation of the anionic state is essentially irreversible, forming the corresponding dienoyl-CoA compounds. In the case of 3-Cl-BzCoA, but not 3-F-BzCoA, the halogenated dienoyl-CoA spontaneously eliminates HCl in an E2 manner. During conversion of 4-F-BzCoA, the pK_a_ of the assumed 4-F-dienoyl-CoA intermediate (C-3 position) is significantly decreased. As a result, the essentially irreversible fluoride release in an E1cB-type elimination is driven by rearomatization and favored over 4-F-dienoyl-CoA formation.

The results obtained in this work demonstrate the functional versatility of class I BCRs in the anaerobic catabolism of various aromatic compounds. Next to the originally described role as central dearomatizing enzyme (23, 28, 29), class I BCRs catalyze dehalogenation reactions during the complete degradation of brominated, chlorinated, and fluorinated aromatics. The reductions of BzCoA, 3-Br-BzCoA, 3-Cl-BzCoA, and 4-F-BzCoA during the complete degradation of the corresponding halogenated aromatic acids and other monocyclic compounds are likely all initiated by similar electron transfer and protonation events yielding a common anionic state (32) (Fig. 6). Depending on the position and nature of substituents on the aromatic ring, three different scenarios may occur for the subsequent conversion of the anion. (i) During BzCoA dearomatization, the anion is protonated at the C-3 position in an essentially irreversible manner, yielding dienoyl-CoA (24, 32). (ii) In the case of 3-Cl-/3-Br-BzCoA reduction, the same protonation yields the 3-Cl-/3-Br-dienoyl-CoA intermediates that are prone to spontaneous one-step E2 elimination of HCl/HBr, driven by rearomatization (26). Likewise, conversion of 3-F-BzCoA yields 3-F-dienoyl-CoA; however, due to the strength of the C–F bond, HF cannot be eliminated. Consequently, the 3-F-dienoyl-CoA represents a dead-end product (26). (iii) During conversion of 4-F-BzCoA, a possible protonation of the anionic state would yield a 4-F-dienoyl-CoA intermediate with an unusual low pK_a_ of 13.4 (calculated by MarvinSketch software version 15.4.6.0; ChemAxon). As a result, the reversible protonation of the anionic transition state is less favored than the essentially irreversible C–F bond cleavage driven by rearomatization to BzCoA. In the case of 3-F-dienoyl-CoA, the pK_a_ of the proton at C-4 is 29.4 and therefore far too high for an HF elimination scenario. The conversion of 4-F-BzCoA to BzCoA plus HF formally corresponds to an aromatic nucleophilic substitution of a fluoride by a hydride with the latter being formed by two consecutive single-electron transfer steps and a protonation step according to the suggested Birch-like reduction (32). Nevertheless, assuming that the proposed anionic state is in equilibrium with 4-F-dienoyl-CoA, 4-F-BzCoA reduction could also be regarded as an E1cB-type elimination of HF from a 4-F-dienoyl-CoA intermediate. The competition between fluorine abstraction and protonation of the assumed anionic transition state during the chemical Birch reduction of fluorobenzenes has been described using hydrated electrons (H_2_O)_n_^−^ and fits well with the proposed mechanism of enzymatic 4-F-BzCoA defluorination (33). We suggest that the inability to abstract fluorine from 3-F-BzCoA by class I BCR is the reason why biodegradation of 3-F-benzoate has never been observed under anoxic conditions.

In a previous work, 4-F-BzCoA has been reported to act as a competitive inhibitor but not as a substrate for isolated class I BCR (24). This previously overlooked reductive 4-F-BzCoA reduction by class I BCR may be explained by the low conversion rate (6% compared to BzCoA), and the rather insensitive spectrophotometric assay used in the previous study. During growth of *T. aromatica* with 4-F-benzoate, this low intrinsic activity is partially compensated by a transcriptional upregulation of the *bcrABCD* genes by a factor of four resulting in a specific 4-F-BzCoA reduction activity of around 25 to 30% in extracts of cells grown with 4-F-benzoate compared to BzCoA reduction activity in extracts of cells grown with benzoate. This finding perfectly fits to the observed differing growth rates with 4-F-benzoate and benzoate and indicates that conversion of 4-F-BzCoA into BzCoA plus HF is the rate-limiting step of 4-F-benzoate catabolism. In parallel, transcription of the *bcl* gene encoding BCL, that shows equal activities with benzoate and 4-F-benzoate, was downregulated by a factor of four. This finding can be interpreted as a response to the lower activity of BCR with 4-F-BzCoA compared to BzCoA. Downregulation of *bcl* gene transcription avoids massive 4-F-BzCoA accumulation that would exhaust the cellular CoA pool; for this reason, we interpret the observed regulatory process as essential for growth with 4-F-benzoate.

The transcriptional up- and downregulation of the genes encoding BCR and BCL during growth with 4-benzoate versus benzoate is remarkable. The observed regulation may be accomplished by a 4-F-benzoate sensing system that is capable of distinguishing between fluorinated and nonfluorinated aromatic growth substrates. As a probably more likely alternative, the regulatory system responds to differential cellular aromatic thioester concentrations. For example, when switching from benzoate to 4-F-benzoate as the growth substrate, the high activity of BCL with 4-F-benzoate and the relatively low activity of BCR with 4-F-BzCoA will immediately result in accumulation of 4-F-BzCoA. Increased 4-F-BzCoA concentrations could then negatively affect *bcl* transcription and stimulate *bcrABCD* transcription. The observed reciprocal regulation of gene expression would differ from the previously suggested (34) and experimentally verified (35) positive effect of BzCoA on both BCL and BCR gene transcription in *Rhodopseudomonas palustris* (34) and *Azoarcus* strain CIB (35).

## MATERIALS AND METHODS

### Growth of bacterial cells and preparation of cell extracts. 

*T. aromatica* strain K172 (DSM 6984) was grown anaerobically at 30°C in a mineral salt medium as described previously (14). Benzoate/4-F-benzoate (2.7 mM) and toluene/4-F-toluene (2 mM) were used as the sole sources of carbon and nitrate as the terminal electron acceptor (12.7 mM or 8 mM, respectively). For growth with toluene, paraffin had been added to the medium to a final concentration of 2% (vol/vol). Growth was monitored by measuring the optical density of cell suspensions in 1-cm cuvettes at 578 nm.

Concentrations of growth substrates were measured in cell-free supernatant after centrifugation of cell suspensions at 10,000 × *g* (4°C, 10 min). Analysis was carried out by reversed-phase high-pressure liquid chromatography (HPLC) using a Waters 2690 separation module. It was combined with a Eurospher 100-5 C_18_ column (250 by 4 mm) (Knauer) that was equilibrated with 40 mM formic acid containing 9% methanol at a flow rate of 1 ml min^−1^. Separation was achieved by applying a rising gradient of methanol to 45% within 2 min and further to 81% within 6 min. Products were identified by comparing retention times and UV/Vis absorption spectra with standards.

Harvesting of cells was carried out by centrifugation in the exponential growth phase at 4,500 × *g* (4°C, 15 min) followed by immediate further processing or storage in liquid nitrogen. Cell extracts were prepared under anaerobic conditions in 20 mM triethanolamine hydrochloride (TEA)/KOH buffer (pH 7.8) (1 g of cells in 1 ml of buffer), containing 4 mM MgCI_2_, 10% glycerol (vol/vol), 1 mM dithioerythritol (DTE), 100 µM Na_2_S_2_O_4_, and 0.1 mg DNase I. Subsequent French press treatments at 82 bars were followed by centrifugation at 100,000 × *g* (4°C, 75 min).

### Cell suspension assays. 

Cells were harvested anaerobically in the mid-exponential growth phase (4,300 × *g*, 4°C, 15 min) and washed twice in mineral salt medium without carbon source by centrifugation. Cells were finally adjusted to an optical density of 9.0 (578 nm) by suspension in a medium containing 12 mM NaNO_3_ in the absence of a carbon source. Reactions were started by adding individual carbon substrates to a final concentration of 0.8 mM and stopped by adding equal volume amounts of 20% (vol/vol) formic acid. Samples were centrifuged at 18,000 × *g* (4°C, 10 min) prior to HPLC analysis.

### Synthesis and analysis of CoA thioesters. 

BzCoA was synthesized from benzoic acid anhydride and CoA (36). 4-F-BzCoA was synthesized starting from the corresponding acid via its succinimide ester as described previously (37). All products were purified by preparative reversed-phase HPLC as described previously (37). The extinction coefficient of 4-F-BzCoA was determined as ε_260_ = 28,270 M^−1^ cm^−1^ by determination of free thiol groups after alkaline hydrolysis. CoA ester hydrolysis was performed at pH 12 by incubation at 80°C for 10 min. After neutralization with HCl, the concentration of CoA was determined by the method of Ellmann, assuming an extinction coefficient ε_412_ = 14,150 M^−1^ cm ^− 1^ (38).

Chromatographic analysis of CoA thioesters was performed by reversed-phase ultraperformance liquid chromatography (UPLC) on an Acquity H-class system combined with a BEH C_18_ column (1.7-µm; 2.1 × 100 mm) (Waters). Separation was achieved by applying an increasing gradient of acetonitrile in 10 mM potassium phosphate buffer (pH 6.8) at a flow rate of 0.2 ml min^−1^. Starting at 2%, the acetonitrile concentration was immediately increased to 20% and further to 25% within 7.8 min. Products were identified by comparing retention times and UV/Vis absorption spectra with standards.

### Ion chromatography. 

The concentrations of released fluoride during growth with 4-fluorobenzoate were determined in cell-free supernatant after centrifugation of cell suspensions at 10,000 × *g* (4°C, 10 min). Measurements were performed by using a Dionex ICS-2100 ion chromatography system with Dionex IonPac AS11-HC column (analytical column; 2 mm by 250 mm). The samples (10 µl) were analyzed isocratically by using 5 mM KOH at a flow rate of 0.38 ml min^−1^. Fluoride was detected by suppressed conductivity with a retention time of 2.7 min. NaF dissolved in water was used as a calibration standard at different concentrations.

### Purification of defluorinating BCR. 

Purification was performed based on a previously described method (23); all modifications are stated in the following.

All buffers contained 50 µM sodium dithionite. Cell extract obtained from 5 g of cells (wet weight) grown with 4-fluorobenzoate was applied to a DEAE-Sepharose column (fast flow, GE Healthcare; diameter, 26 mm; volume, 16 ml) at a flow rate of 0.5 ml min^−1^. Further steps were performed at 1 ml min^−1^. Fractions exhibiting defluorination activity were pooled and transferred to a ceramic hydroxyapatite column (BioRad; diameter, 16 mm; volume, 5 ml) at a flow rate of 1 ml min^−1^. Pooled protein was subsequently loaded to a Source 15Q column (GE Healthcare; diameter, 16 mm; volume, 6 ml) at 1 ml min^−1^. The column had been equilibrated with 20 mM triethanolamine/Cl buffer (pH 7.3), 4 mM MgCl_2_, and 10% (vol/vol) glycerol. After the column was washed with 90 mM KCI in buffer (5 bed volumes), a linear gradient from 90 to 130 mM KCl in buffer was applied over 10 bed volumes. Pooled 2-ml fractions were concentrated by a factor of 140 using Vivaspin Turbo 4 centrifugal concentrators (Sartorius) (pore exclusion size of 30 kDa; 4 ml). Two hundred fifty microliters of the concentrated solution were applied at a flow rate of 0.1 ml min^−1^ to a fast protein liquid chromatographic (FPLC) Superdex 200 10/300 GL column (GE Healthcare) which had been equilibrated as described previously (23); for separation, the flow rate was 0.3 ml min^−1^.

### Determination of enzyme activities. 

The ATP-dependent reduction of BzCoA or 4-F-BzCoA catalyzed by enriched BCR were determined at 30°C using a continuous spectrophotometric assay with dithionite reduced methyl viologen serving as the electron donor as described before (23). For activity determination in cell extracts and the identification of CoA-ester intermediates, a discontinuous HPLC assay was applied as described earlier (30). Analysis was routinely performed by C_18_ reversed-phase UPLC as described above. For MS analyses, collected compounds were freeze-dried and dissolved in water before analysis on an API 2000 ESI-MS/MS triple quadrupole mass spectrometer (Sciex Applied Biosystems) coupled to an Agilent 1100 HPLC system. A solvent mixture of 20% ammonium acetate and 80% acetonitrile at a flow rate of 0.1 ml min^−1^ was applied. Measurements were carried out in positive-ion mode (Q1) with a TurboSpray ion source and a scan range of *m/z* 300 to 900 after direct injection of samples.

For analysis of BCL activity, **s**oluble protein fractions were precipitated with saturated ammonium sulfate solution and aromatic carboxylic acid CoA ligase activities were determined at 30°C using a coupled continuous spectrophotometric assay as described previously (39).

### Quantitative reverse transcription PCR. 

Cells were anaerobically harvested in the mid-exponential growth phase. Total RNA was isolated using a Direct-zol RNA miniprep kit (Zymo Research) and DNase I treated using a Turbo DNA-free kit (Thermo Fisher Scientific). RNA concentration, purity, and integrity were determined with a NanoDrop 1000 spectrophotometer (Peqlab) and sodium hypochlorite agarose gels. Reverse transcription of 300 ng RNA was performed using a RevertAid first strand cDNA synthesis kit (Thermo Fisher Scientific) with random hexamer primers. For quantitative PCR (qPCR) analysis, cDNA was diluted 20-fold (vol/vol) in nuclease-free H_2_O and added to SsoAdvanced SYBR green supermix (Bio-Rad) containing 250 nM (each) gene-specific primers (see Table S3 in the supplemental material). Primers were designed with Primer3 (40), and specificities were validated using BLAST. qPCR was performed on a CFX96 touch real-time PCR cycler (Bio-Rad). Among a total of four putative reference genes, the genes encoding the bacterial actin homologue MreB and the alpha subunit of a nitrate reductase Z were identified as suitable reference genes with the software program qBase+ (Biogazelle) using the geNorm algorithm (41). Transcription of target genes was normalized (42) on both reference genes using qBase+.

### Proteomic analysis by mass spectrometry. 

Forty micrograms of protein was precipitated with ice-cold acetone (5 volumes) for 30 min at −20°C followed by centrifugation at 12,000 × *g* and 4°C for 10 min. After careful removal of the supernatants, the protein pellets were resuspended in 2× sodium dodecyl sulfate (SDS) lysis buffer, incubated at 90°C for 5 min, and centrifuged at 1,000 rpm. Protein samples were loaded on an SDS-polyacrylamide gel, run for 5 to 10 min at 10 to 20 mA (short separation), and afterward stained with Coomassie blue G-250 (Merck, Darmstadt, Germany). Proteolytic in-gel digestion with trypsin was performed as described previously (43). Proteolytic peptide mix of bovine serum albumin (Sigma Aldrich) (100 fmol) was added to each sample as an internal standard.

Proteome analyses were performed using an Orbitrap Fusion mass spectrometer (Thermo Fisher Scientific, Waltham, MA, USA) coupled to a TriVersa NanoMate (Advion, Ltd., Harlow, United Kingdom). In total, 5-µl volumes of the peptide lysates were separated with a Dionex UltiMate 3000 nano-LC system (Dionex/Thermo Fisher Scientific, Idstein, Germany). Further details are listed in Table S4 in the supplemental material. MS raw files were processed using Proteome Discoverer (v1.4; Thermo Fisher Scientific). MS spectra were searched against a database containing the UniProt sequences of bovine serum albumin, *Thauera aminoaromatica* and *Thauera aromatica* K172 (4,034 sequence entries) using the SEQUEST algorithm. Enzyme specificity was selected to trypsin with up to two missed cleavages allowed using 5 ppm MS tolerance and 0.1 Da MS/MS tolerances. Oxidation (methionine) and acetylation (lysine) were set as dynamic modifications, and carbamidomethlyation (cysteine) was selected as a fixed modification. Peptide spectrum matches (PSMs) were validated using percolator with a false-discovery rate (FDR) of <1% and quality filtered for XCorr of ≥2.25 [+2] and ≥2.5 [+3]. Protein quantification was done with the Precursor ion area detector (2 ppm mass precision) of Proteome Discoverer. The abundance of one identified protein was quantified by the average abundance of the top three peptides assigned to this protein. The protein area was further log_2_ transformed, and the median values were normalized in order to compare with the abundance of other proteins. The differential protein abundance levels of BCL and BCR during growth with 4-F-benzoate versus benzoate are presented by relative fold changes.

## SUPPLEMENTAL MATERIAL

Figure S1  Growth of *T. aromatica* K172 with 4-fluorotoluene (○) measured by OD_578_ and growth of *T. aromatica* K172 with the indicated concentrations of free fluoride (×). The means of two biological replicates are shown. The doubling time was 16.2 h. Download Figure S1, PDF file, 0.2 MB

Table S1  Determination of 4-F-benzoate consumption, fluoride release, and growth yield at different time points during anaerobic degradation of 4-F-benzoate by *T. aromatica* Table S1, PDF file, 0.2 MB

Table S2  Data set of proteins identified. Table S2, XLSX file, 0.4 MB

Table S3  Oligonucleotide primers used for quantitative reverse transcription-PCR analyses. Table S3, PDF file, 0.2 MB

Table S4  Parameters of LC-MS analyses of proteomes. Table S4, PDF file, 0.3 MB
