# Supplementary material for: ATP-Dependent C–F Bond Cleavage Allows the Complete Degradation of 4-Fluoroaromatics without Oxygen
Source: mBio. 2016 Aug 9;7(4):e00990-16. doi: 10.1128/mBio.00990-16 (PMC4992971; doi:10.1128/mBio.00990-16)
Supplement: Figure S1 — Growth of T. aromatica K172 with 4-fluorotoluene (○) measured by OD578 and growth of T. aromatica K172 with the indicated concentrations of free fluoride (×). The means of two biological replicates are shown. The doubling time was 16.2 h. Download [file mbo004162938sf1.pdf]

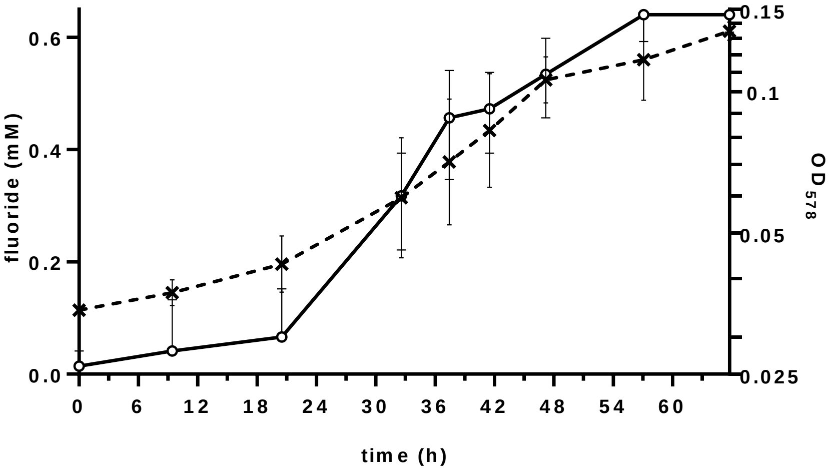


**Supplementary Figure S1** Growth of *T. aromatica* K172 with 4-fluorotoluene (**—O—**) $OD_{578}$, (--**x**--) concentration of free fluoride; number of biological replicates: 2; doubling time 16.2 h
