# Supplementary material for: ATP-Dependent C–F Bond Cleavage Allows the Complete Degradation of 4-Fluoroaromatics without Oxygen
Source: mBio. 2016 Aug 9;7(4):e00990-16. doi: 10.1128/mBio.00990-16 (PMC4992971; doi:10.1128/mBio.00990-16)
Supplement: Table S1 — Determination of 4-F-benzoate consumption, fluoride release, and growth yield at different time points during anaerobic degradation of 4-F-benzoate by T. aromatica [file mbo004162938st1.pdf]

Supplemental Table S1 Determination of 4-F-benzoate consumption, fluoride release and growth yield at different time points during anaerobic degradation of 4-F-benzoate by *T. aromatica*.

time (h)	4-F-benzoate consumed (mmol)	fluoride released (mmol)	growth yield (g cells [dry weight] per mol 4-F-benzoate consumed)
6,7	19,7	19,1	33,3
9,2	41,7	39,4	33,7
11,8	60,8	49,1	35,4
13,3	69,0	87,2	41,2
22,0	229,3	219,5	35,6
24,5	267,6	244,4	35,4
