# Supplementary material for: ATP-Dependent C–F Bond Cleavage Allows the Complete Degradation of 4-Fluoroaromatics without Oxygen
Source: mBio. 2016 Aug 9;7(4):e00990-16. doi: 10.1128/mBio.00990-16 (PMC4992971; doi:10.1128/mBio.00990-16)
Supplement: Table S3 — Oligonucleotide primers used for quantitative reverse transcription-PCR analyses. [file mbo004162938st3.pdf]

Supplementary Table S3 Oligonucleotide primers used for quantitative RT-PCR analyses.

**Primer pair 5'→3'**	**Target annotation**
AGCGTGCGAAGAAAATC<br>TCGACGACCTTCTTCTC	F0F1 ATP synthase beta subunit (housekeeping control 1)
ACGTAGATCAGGGTGTTG<br>GTTTCCTGCGCTCTTAT	rod shape-determining protein MreB (housekeeping control 2)
TGGTGGAGCTGTAGTAGATA<br>AGGTCAGCATCTTCCAT	succinate dehydrogenase flavoprotein subunit (housekeeping control 3)
ACTTCTTGAGCTCTTCGTAC<br>GACTACAGCCAGATCTACAA	nitrate reductase Z alpha subunit (housekeeping control 4)
AACTTCCAGATGAACGAC<br>GACTTGGTCGACTTCATC	benzoyl-CoA reductase alpha subunit
CATGAAGGAAGAGAAGTACC<br>GTAGAACAGCTTCCAGAACT	benzoyl-CoA reductase beta subunit
CATGAAGGAAGAGAAGTACC<br>GTAGAACAGCTTCCAGAACT	benzoyl-CoA reductase gamma subunit
CATGAAGGAAGAGAAGTACC<br>GTAGAACAGCTTCCAGAACT	benzoyl-CoA reductase delta subunit
AGATTCCGGAGCGCTACAAC<br>CATCGTAGGTGTAGGAGCCG	benzoate:CoA ligase
