# Supplementary material for: ATP-Dependent C–F Bond Cleavage Allows the Complete Degradation of 4-Fluoroaromatics without Oxygen
Source: mBio. 2016 Aug 9;7(4):e00990-16. doi: 10.1128/mBio.00990-16 (PMC4992971; doi:10.1128/mBio.00990-16)
Supplement: Table S4 — Parameters of LC-MS analyses of proteomes. [file mbo004162938st4.pdf]

Supplemental Table S4 Parameters of LC-MS analyses of proteomes.

**Reversed phase liquid chromatography (RP-LC)**	
Instrument	Ultimate 3000 RSLC (Dionex, Idstein, Germany)
trap column	75 µm inner diameter, packed with 3 µm C18 particles (Acclaim PepMap100, Thermo Scientific)
analytical column	15 cm analytical column packed with 2 µm C18 particles (Acclaim PepMap RSLC, Thermo Scientific)
buffer system	binary buffer system consisting of 0.1% formic acid, 2% ACN (buffer A) and 0.08% formic acid in 80% ACN in (buffer B)
flow rate	300 nl/min
Gradient	linear gradient of buffer B from 4% up to 55%
gradient duration	60 min
column oven temperature	35°C

**Mass spectrometry (MS)**	
instrument	Orbitrap Fusion mass spectrometer (Thermo Scientific)
electrospray	via TriVersa NanoMate (Advion Biosciences, Norwich, UK)
operation mode	data-dependent
MS scan resolution	120,000
MS ion target value	4e5
maximum ion injection time for the MS scan	60 ms
selection for MS/MS	Top Speed 3 sec cycle time - most intense isotope patterns with charge ≥2 from the survey scan
isolation window	1.6 *m/z*
dissociation mode	higher energy collisional dissociation (HCD)
normalized collision energy	28%
maximum ion injection time for the MS/MS scans	150 ms
MS/MS ion target value	50,000
dynamic exclusion	30 s
MS/MS operation mode	centroid mode
